# In Vitro Study of Mutagenesis Induced by Crocidolite-Exposed Alveolar Macrophages NR8383 in Cocultured Big Blue Rat2 Embryonic Fibroblasts

**DOI:** 10.1155/2010/323828

**Published:** 2010-06-07

**Authors:** Yves Guichard, Laurent Gaté, Christian Darne, Marie-Claire Bottin, Cristina Langlais, Jean-Claude Micillino, Michèle Goutet, Schmit Julien, Binet Stéphane

**Affiliations:** Institut National de Recherche et de Sécurité, rue du Morvan CS 60027, 54519 Vandoeuvre-Les-Nancy Cedex, France

## Abstract

Asbestos-induced mutagenicity in the lung may involve reactive oxygen/nitrogen species (ROS/RNS) released by alveolar macrophages. With the aim of proposing an alternative in vitro mutagenesis test, a coculture system of rat alveolar macrophages (NR8383) and transgenic Big Blue Rat2 embryonic fibroblasts was developed and tested with a crocidolite sample. Crocidolite exposure induced no detectable increase in ROS production from NR8383, contrasting with the oxidative burst that occurred following a brief exposure (1 hour) to zymosan, a known macrophage activator. In separated cocultures, crocidolite and zymosan induced different changes in the gene expressions involved in cellular inflammation in NR8383 and Big Blue. In particular, both particles induced up-regulation of iNOS expression in Big Blue, suggesting the formation of potentially genotoxic nitrogen species. However, crocidolite exposure in separated or mixed cocultures induced no mutagenic effects whereas an increase in Big Blue mutants was detected after exposure to zymosan in mixed cocultures. NR8383 activation by crocidolite is probably insufficient to induce in vitro mutagenic events. The mutagenesis assay based on the coculture of NR8383 and Big Blue cannot be used as an alternative in vitro method to assess the mutagenic properties of asbestos fibres.

## 1. Introduction

Asbestos forms a group of naturally occurring mineral fibres (defined as having a ≥3 : 1 length to diameter ratio) that are associated with the development of both malignant (cancer, mesothelioma) and nonmalignant (asbestosis) diseases of the lung and pleura [1]. Mechanisms of asbestos-induced carcinogenesis are thought to be multiple, including generation of reactive oxygen (ROS) and nitrogen species (RNS), alteration of mitochondrial function, physical disturbance of cell cycle progression, and activation of several signal transduction pathways [2, 3]. The diversity of the many putative pathways has raised a challenge in the development of in vitro models that represent the actual in vivo progress of asbestos carcinogenesis. In vitro studies have shown that asbestos fibres are cytotoxic and clastogenic but not mutagenic in Ames assays. Earlier attempts to define the direct mutagenic potential of asbestos fibres at a genomic locus, such as the hprt gene in a variety of mammal cells, have yielded negative results [4]. Mutagenic assays suitable for detecting either large deletion or homologous recombination have been used to show the mutagenic potential of various fibre types. They suggest that direct exposure of cells to asbestos induces major deletions rather point mutations [5, 6]. However, these genotoxicity data may not reflect every possible effect of in vivo exposure, particularly indirect genotoxicity, which is related to the production of DNA reactive radicals (ROS or RNS) via secondary mechanisms [7]. Recently, several studies using transgenic mutational assays (detecting in vivo point mutation but not clastogenic effects) in fibre genotoxicity testing have reported gene mutations induced by asbestos fibres [8–11]. Crocidolite was shown to induce a 2-fold transient increase in mutant frequencies in mouse lung DNA 4 weeks after nose-only inhalation [9]. Another study using transgenic Big Blue rats demonstrated that crocidolite exposure increases both mutant frequencies and the level of 8-hydroxy-2′deoxyguanosine (8-OHdG) in omentum major DNA. The main form of mutation found in this study was G to T, which is known to be induced by 8-OhdG. This suggests involvement of ROS and RNS in crocidolite-induced mutagenesis in vivo [11]. 

ROS and RNS can be generated through the presence of iron on the fibre surface. However, there is some evidence that asbestos-exposed pulmonary macrophages and neutrophils release ROS/RNS during phagocytosis. Furthermore, asbestos-exposed macrophages release various inflammatory cytokines, such interleukin 1 (IL1) and the tumor necrosis factor-alpha (TNF-*α*). These proteins are assumed to be involved in pulmonary fibrotic processes occurring during asbestosis as they increase the activation of neutrophils and fibroblasts [12, 13]. In addition, cytokine and oxidative stress can increase the expression and activity of the inducible nitric oxide synthase (iNOS) in pulmonary alveolar epithelial cells [14]. This enzyme is responsible for the formation of nitric oxide (NO), a precursor of peroxynitrite, which is considered cytotoxic and genotoxic. Asbestos exposure can also affect the balance of oxidant and antioxidant factors in lung cells, such as superoxide dismutase (SOD) [15]. 

Coculture systems of phagocytes and epithelial cells have been described for mineral-fibre related toxicity or inflammation studies [16–19]. Using primary human blood monocytes cocultivated separately with bronchial epithelial cells, Kienast et al. demonstrated that chrysotile phagocytosis resulted in the release of ROS in monocytes and induced DNA single-strand breaks in bronchial epithelial cells [17]. In a previous study, chrysotile phagocytosis was shown to induce the release of interleukin 1-*β* (IL1-*β*), interleukin 6 (IL6), and tumor necrosis factor-alpha (TNF-*α*) in both cocultivated cell types [20]. 

The aim of the study presented here was to investigate whether a coculture system of rat alveolar macrophage cells (NR8383) and transgenic Big Blue Rat2 embryonic fibroblasts (Big Blue cells) can be used to assess the mutagenic potential of mineral fibres. This model was tested with a crocidolite sample known to induce genomic point mutations in vivo [9]. The following endpoints were assessed: ROS generation from NR8383, changes in the gene expression levels of inflammatory factors (IL1-*β*, IL6, TNF-*α*, iNOs, and SOD) in the two cocultivated cells and mutant formation in Big Blue cells. Zymosan was used as a particulate stimulant known to activate NR8383 in the form of an oxidative burst [21]. 

## 2. Materials and Methods

### 2.1. Cell Culture

Big Blue Rat2 embryonic fibroblast (Stratagene, La jolla, CA) and alveolar macrophage-derived NR8383 (American Type Culture Collection, Manassas, VA) cell lines were cultured at 37°C, 5% CO_2_, in a complete medium (Dulbecco's modified Eagle's medium, Invitrogen, France) containing 10% foetal bovine serum (Dutscher, France), 2 mM L-glutamine (Invitrogen), and antibiotics (50 units/mL Penicillin, 50 *μ*g/mL Steptomycin, Invitrogen). Under these conditions, population doubling times for Big Blue and NR8383 monocultures were 24 and 72 hours, respectively. Separated cocultures were produced using cell culture insert systems (Dutscher). Big Blue cells were added to the lower compartment, and NR8383 cells were placed in the upper compartment incorporating 0.4 *μ*m pore size, polyethylene terephttalate membranes. Monocultures and mixed cocultures were produced in 12-well tissue culture plates (Dutscher). For mixed cocultures, Big Blue cells were added to the wells first, followed by NR8383 cells after complete attachment of Big Blue cells. Cell cultures were prepared one day prior to chemical exposure. Big Blue/NR8383 cell ratios in mixed and separated cocultures were 1 : 2 and 1 : 6, respectively, so that cell cultures were 80% confluent at the end of the treatment period. In order to avoid misinterpretation of results in the mutagenesis assay, cell mortality in control and treated cultures was measured using the Trypan-Blue exclusion method. The treatment conditions used in this study all induced less than 20% cell mortality in the two cell types.

### 2.2. Fibres and Particles Preparation

The crocidolite sample used in this study was donated by R.E.G. Rendall (National Center for Occupational Health, Johannesburg, South Africa). Physical chemical analysis of this crocidolite batch has been extensively described in previous studies [9, 22]. Size distributions based on fibre length (L) and diameter (D) were 83.1% for *L* < 5 *μ*m and *D* < 3 *μ*m and 16.9% for *L* > 5 *μ*m and *D* < 3 *μ*m. The number of particles per unit of mass was estimated to be 16 × 10^5^ fibres/*μ*g. On the day of cell treatment, crocidolite and zymosan particles (Sigma, France) were suspended in complete medium. Serial dilutions, thoroughly mixed each time, were added to the cell cultures.

### 2.3. Chemiluminescence Assay

The chemiluminescence assay used lucigenin, a specific probe for superoxide anion (O_2_^−^) [23]. This assay has been previously described in relation to measuring NR8383 ROS production after exposure to particles, including zymosan [24]. NR8383 cell suspensions in complete medium were added to a 96-well plate (2 × 10^5^ cells per well) and allowed to adhere to the plate for approximately 3 hours. Lucigenin (Sigma, France), prediluted in complete medium (54 *μ*M final concentration), and suspensions of crocidolite (final concentrations of 15, 30, and 60 *μ*g/cm^2^) or zymosan (7.5, 15, and 30 *μ*g/cm^2^ final concentration) were sequentially added to the wells. Chemiluminescence was measured every 5 minutes for 2 hours at 37°C using a Synergy HT (Biotek, France) plate reader.

### 2.4. Fluorescence Assay

A 2′,7′-dichlorofluorescein diacetate (H_2_DCFDA) nonfluorescent dye is capable of passively entering a cell at the location where cellular esterases hydrolyse its acetyl moieties. Here, the probe is susceptible to reaction with a variety of ROS, including hydrogen peroxide, peroxyl radicals, and peroxynitrite anions [25]. Fluorescence of DCF in NR8383 was measured by flux cytometry based on a previously described method [26]. H_2_DCFDA (Invitrogen), prediluted in complete medium (25 *μ*M final concentration), was added to the NR8383 culture in a 12-well tissue culture plate for 30 minutes at 37°C. Suspended crocidolite (1, 5, and 10 *μ*g/cm^2^) or zymosan (10 *μ*g/cm^2^) was then added to the wells and the cultures subsequently exposed for 3 hours. After exposure, the cells were recovered by scraping, then washed and re-suspended in HBSS. Propidium iodide (Sigma) was added to the cell suspensions (50 *μ*g/mL final concentration) and the relative mean fluorescence of green DCF fluorescence within live cells (at least 15,000 cells) measured using a flow cytometer (FACStar Plus-Becton Dickinson, France).

### 2.5. TNF-*α*, IL1-*β*, IL6, iNOS, and SOD2 Gene Expression

Gene expression in NR8383 and Big Blue cells was investigated in separated cocultures by the quantitative RT-PCR method, with *β*-actin used as the housekeeping gene. Table 1 details the primers used. NR8383 cells were exposed to crocidolite or zymosan (15, 30, and 60 *μ*g/cm^2^ final concentrations) for 3 hours. Crocidolite exposure was extended to 24 hours but at lower final concentrations (3 and 15 *μ*g/cm^2^) in order to reduce cytotoxic effects. At the end of the exposure period, the cocultivated cells were trypsinized and collected by centrifugation. Total RNA was extracted from the cells using a Stratagene Absolutely RNA RT-PCR Miniprep kit, including the DNAse I digestion step in accordance with the manufacturer's instructions. RNA quality was determined with a 1.5% agarose gel, and concentrations were measured using a Beckman DU 640 B spectrophotometer (Beckman Coulter, France). RNA (500 ng) was reverse transcribed with 500 ng of oligo(dT)_12–18_ using the SuperScript II RT (50 U) (Invitrogen) following the manufacturer's testing procedure. As a negative control, a sample containing RNA but without the RT enzyme was also included. Quantitative RT-PCR was performed with a LightCycler (Roche, France) using the QuantiTect SYBR Green PCR kit (Qiagen, France). In brief, 25 ng of reverse-transcribed RNA were mixed with 5 pmol of specific primers for a given gene and the QuantiTect SYBR Green PCR mix. PCR amplification was performed for 15 minutes at 95°C for *n* cycles [15 seconds at 95°C, 20 seconds at *x*°C, and 15 seconds at 72°C], where the values of *n* and *x* depended on the primer set and the targeted gene. The relative quantity of each mRNA was determined using the Pfaffl model [27]. For each gene, a standard curve was plotted and its slope used to calculate the efficiency (*E*) of the PCR reaction (*E* = 10^[−1/slope] ^). For each sample, the relative expression of a given gene was calculated from the threshold cycle (CT) value, which is the number of cycles for which a statistically significant increase in PCR product is first detected. The fold change of a target gene is expressed as exposed cells with respect to control cells, compared to *β*-actin (used as a reference). *E*_target_ is the real-time PCR efficiency of the target gene transcript; *E*_*β*actin_ is the real-time PCR efficiency of the *β*-actin transcript

(1) $$\text{Fold change}=\frac{\left[{{{\left(E_{\text{target}}\right)}^{\Delta \text{CT}}}_{\text{target}}}^{\left(\text{control}-\text{exposed}\right)}\right]}{\left[{{{\left(E_{\beta \text{actin}}\right)}^{\Delta \text{CT}}}_{\beta \text{actin}}}^{\left(\text{control}-\text{exposed}\right)}\right]}.$$

### 2.6. Mutagenesis Assay

The *cII* assay in the Big Blue system allows positive selection of mutant cells in vivo and in vitro [28, 29]. The mutagenesis assay was validated in Big Blue monocultures with N-ethyl-N-nitrosourea (ENU) which is a well characterized mutagen [30]. Before treatment, cultures were washed three times in Hank's balanced salt solution (HBSS, Invitrogen) and received ENU solution (Sigma, France) prediluted in HBSS for 30 minutes (100 and 500 *μ*g/mL final concentration). Crocidolite treatments were performed on both separated and mixed cocultures. In separated cocultures, NR8383 cells were exposed to crocidolite (3 and 15 *μ*g/cm^2^) for 72 hours. Because crocidolite induces more cytotoxicity in mixed than in separated cocultures, exposure of mixed cocultures to crocidolite was limited to 24 hours at the highest concentration possible, avoiding cell mortality in excess of 20% (10 *μ*g/cm^2^). Treatment with zymosan was performed only in mixed cocultures (concentrations of 15, 30, and 10 *μ*g/cm^2^ for 3 hours). 

At the end of treatment, Big Blue cells in monocultures or separated cocultures were trypsinized, transferred to 175 cm^2^ tissue culture flasks, and grown for 4-5 days, without exposure, to allow fixing of chemical-induced mutations. In the case of the mixed cocultures, the culture medium was changed 3 hours after transferring the cells to the flasks, and every day for 5 days thereafter, to eliminate the majority of the NR8383 cells. Cells were trypsinized and collected by centrifugation for mutation analysis purposes. Mutant Big Blue cells were assessed using the Lambda Select cII mutagenesis assay (Stratagene), based on the manufacturer's instructions. High molecular weight DNA was prepared from around 10^6^ cells, using a RecoverEase DNA isolation kit (Stratagene). Big Blue cells were gently homogenized with a Dounce, and cell nuclei were collected by short centrifugation. Following protein and RNA hydrolysis, DNA was purified by dialysis against a TE buffer. The Lambda shuttle vectors were recovered from purified DNA by in vitro packaging (Transpack, Stratagene). Control and exposed cell cultures were matched for each packaging reaction. An E. coli G1250 host strain was subsequently infected by in vitro packaged phages. Mutants were selected by 41-hour incubation at 24 ± 0.5°C. Phage titres of packaging reactions were evaluated by mixing a dilution of the packaged phages with the E. coli G1250 host strain and incubating them overnight at 37°C. Mutant plaques were identified and confirmed by phage replating at low density. The cII mutant frequency (MF) was determined by dividing the number of mutant plaques by the total number of plaque forming units (PFU) evaluated for each cell culture.

### 2.7. Statistical Analysis

Each experiment was performed at least three times, and experimental data are given as a mean ± standard deviation (mean ± SD). The statistical significance of differences between groups in each assay was subjected to a Student *t*-test (two sides) based on assumed equal variance.

## 3. Results

### 3.1. ROS Detection

ROS production in crocidolite- or zymosan-stimulated NR8383 was investigated using both chemiluminescence and fluorescence assays. 

**Figure 1:**
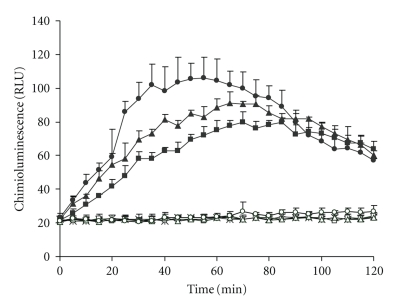
Time course of oxidant production by NR8383 cells after incubation with crocidolite and zymosan. Oxidant production was measured as increased chemiluminescence of lucigenin (in relative light units, RLU). Each point represents the mean ± SD of three experiments. Control (∗); Crocidolite 15 *μ*g/cm^2^ (□), 30 *μ*g/cm^2^ (∆), and 60 *μ*g/cm^2^ (*∘*); Zymosan 7.5 *μ*g/cm^2^ (■), 15 *μ*g/cm^2^ (▲), and 30 *μ*g/cm^2^ (●).

Figure 1 shows lucigenin chemiluminescence measured in crocidolite- and zymosan-exposed NR8383 cultures with respect to time. Exposure of NR8383 cells to various concentrations of crocidolite (15, 30, and 60 *μ*g/cm^2^) elicited no cellular response in the form of superoxide anion production, when compared to the control (unexposed) cultures. In contrast, when NR8383 cells were exposed to zymosan concentrations of 7.5, 15, and 30 *μ*g/cm^2^, a dose-dependant increase in luminescence was observed, indicating superoxide production in stimulated cells. At the highest zymosan concentration, the maximum signal value occurred approximately 1 hour after exposure and resulted in an approximate 5-fold increase in ROS level with respect to the control. 

**Figure 2:**
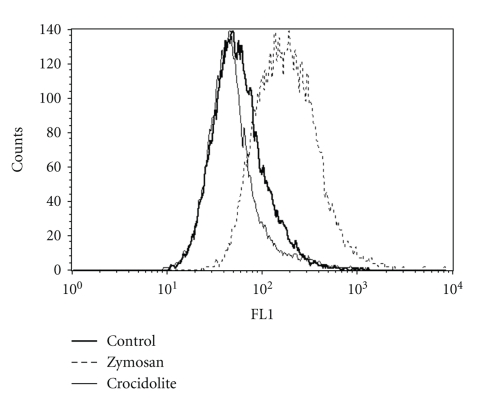
ROS production in NR8383 analysed by flow cytometry. NR8383 cells were preincubated with H_2_DCFDA (25 *μ*m) for 30 minutes and then exposed to 10 *μ*g/cm^2^ of crocidolite or zymosan for 3 hours. Representative histograms plot the relative green DCF fluorescence (FL1) within 15000 live cells (counts: numbers of events).

In the fluorescence assay, NR8383 cells preincubated with H_2_DCFDA were exposed to crocidolite (1, 5, and 10 *μ*g/cm^2^) or zymosan (10 *μ*g/cm^2^) for a period of 3 hours. Higher concentrations of fibres or particles disrupted the flow cytometry analysis. Figure 2 shows typical histograms of DCF fluorescence in NR8383 cells exposed to 10 *μ*g/cm^2^ of crocidolite or zymosan, and Table 2 details the corresponding mean fluorescence values. Compared to the control cells, an approximately 3-fold increase in fluorescence was detected in the zymosan-treated cells, but no significant change was detected in the crocidolite-treated cells (at all concentrations tested), indicating that only zymosan particles stimulated ROS production in NR8383 cells.

### 3.2. Inflammatory Mediator Expression

**Figure 3:**
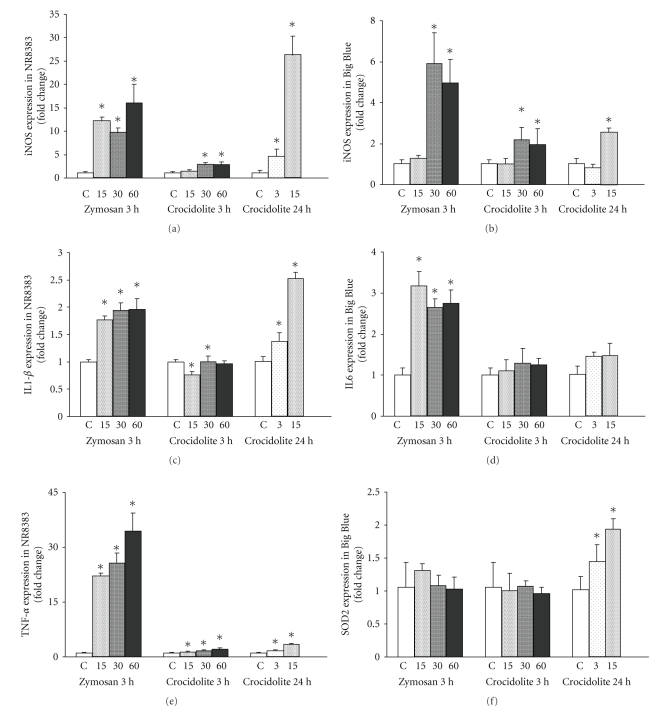
Effect of treatment with crocidolite and zymosan for 3 hours (15, 30, and 60 *μ*g/cm^2^) or with crocidolite for 24 hours (3 and 15 *μ*g/cm^2^) on the expression of various genes in NR8383-Big Blue separated cocultures. Levels of iNOS, IL1*β*, and TNF*α* mRNA in NR8383 and levels of iNOS, IL-6, and SOD-2 mRNA in Big Blue were assessed by qPCR, using *β*-actin as an endogenous external standard. Data are expressed as “fold change” of the control value (see materials and methods). Results represent the mean ± SD of at least 3 experiments. *Statistically significant (*P* < .05) increase in gene expression compared to the control (unexposed) cocultures.

The effects of treatment with crocidolite or zymosan on inflammatory mediator expressions in NR8383 and Big Blue cells were assessed in separated coculture conditions. Cocultures were exposed to crocidolite or zymosan (15, 30, and 60 *μ*g/cm^2^) for 3 hours and to crocidolite (3 and 15 *μ*g/cm^2^) for 24 hours. Figure 3 includes the transcription rates of iNOS, IL1-*β*, and TNF*α* in NR8383 and of iNOS, IL6, and SOD2 in Big Blue.

Three-hour treatment of NR8383 cells with zymosan induced significant increases in mRNA levels of iNOS, IL1-*β*, and TNF*α* with respect to the control (untreated) cocultures: 16.1 ± 4.0-fold, 2.0 ± 0.1-fold, and 34.4 ± 5.0-fold, respectively, at the highest zymosan concentration (60 *μ*g/cm^2^). Under the same treatment conditions, crocidolite induced in NR8383 smaller but significant increases in iNOS and TNF-*α* (2.9 ± 0.5-fold and 2.2 ± 0.3-fold, respectively) but had no significant effect on IL1-*β* mRNA levels. Twenty-four hour exposure to crocidolite induced higher levels of iNOS, IL1-*β*, and TNF-*α* mRNA than 3-hour exposure (26.1 ± 4.2-fold, 2.5 ± 0.1-fold, and 3.4 ± 0.3- fold, respectively, at concentrations of 15 *μ*g/cm^2^).

In cocultivated Big Blue cells, 3-hour treatment with zymosan and crocidolite at 30 and 60 *μ*g/cm^2^ induced significant increases in iNOS mRNA levels. These were higher with zymosan than with crocidolite (5.2 ± 1.2-fold compared to 1.9 ± 0.8-fold, at 60 *μ*g/cm^2^). The level of iNOS expression also increased significantly in Big Blue when cocultures were exposed to crocidolite at 15 *μ*g/cm^2^ for 24 hours (2.5 ± 0.2-fold). Only zymosan-exposed NR8383 induced a significant up-regulation of IL6 expression in Big Blue, corresponding to 2.7 ± 0.3-fold at 60 *μ*g/cm^2^. No change in SOD2 expression in Big Blue was observed after a 3-hour exposure to zymosan or crocidolite. However, 24-hour exposure to crocidolite resulted in a significant increase in SOD2 mRNA levels in Big Blue, corresponding to a 1.9 ± 0.2-fold increase with respect to the control cocultures, at 15 *μ*g/cm^2^ concentration.

### 3.3. Mutagenesis Assay

Mutation frequency (MF) in the *cII* gene was determined in DNA from Big Blue cells following various culture and treatment processes with ENU, crocidolite, and zymosan. At least 3 assays were performed in each treatment group and an average of 300,000 PFU was screened per assay. The effect of treatment on cell mortality was also assessed (cf. Table 3).

Thirty-minute treatments of Big Blue monocultures with ENU concentrations of 100 and 500 *μ*g/mL produced a 4.3-fold (41.8 ± 8.7 × 10^−5^) and an 8.6-fold (83.3 ± 9.8 × 10^−5^) significant increase in MF, respectively, with respect to the control (unexposed) cultures (9.6 ± 1.0 × 10^−5^). 

Crocidolite treatments of separated cocultures (3 and 15 *μ*g/cm^2^ for 72 hours) or mixed cocultures (10 *μ*g/cm^2^ for 24 hours) induced no significant changes in MF compared to the control cocultures. However, 3-hour exposure to zymosan at 15, 30, and 60 *μ*g/cm^2^ in mixed cocultures induced significant increases in MF: 1.4-fold (18.3 ± 2.9 × 10^−5^), 1.3-fold (17.4 ± 1.4 × 10^−5^), and 2.4-fold (30.8 ± 9.8 × 10^−5^) with respect to the control cocultures (13.0 ± 1.5 × 10^−5^). 

ENU and zymosan treatments caused no significant increases in cell mortality at any of the concentrations used in the mutagenesis assays. In separated cocultures, crocidolite treatment at 3 *μ*g/cm^2^ for 72 hours induced no cell mortality in NR8383 and Big Blue. At 15 *μ*g/cm^2^, the treatment induced a significant increase in NR8383 mortality (19.6 ± 1.3% compared to 5.4 ± 2.3% in the control cocultures) but had no significant effect on Big Blue mortality. In mixed cocultures, crocidolite treatment (10 *μ*g/cm^2^ for 24 hours) induced a significant increase in cell mortality (15.0 ± 1.4% compared to 8.2 ± 0.9% in the control cocultures).

## 4. Discussion

The capacity of asbestos fibres to induce ROS production in alveolar macrophages has been reported in different in vivo and in vitro studies [31–33]. Phagocytosis of asbestos fibres by macrophages is often associated with the oxidative burst mediated by the reduced NADPH oxidase system. If “frustrated”, fibre phagocytosis is believed to generate large amounts of reactive oxygen intermediates including superoxide anion, hydroxyl radicals, and hydrogen peroxide [14, 34]. Asbestos fibres can also induce NO and peroxynitrite in rat alveolar macrophages [35, 36]. ROS and RSN are able to induce oxidation or nitrosylation reactions with DNA which are believed to be involved in asbestos-induced mutagenesis [7]. In addition to generating oxidants, asbestos phagocytosis induces stimulation of transcription and secretion of cytokine, for example, IL1*β* and TNF*α* in alveolar macrophages. Both oxidants and cytokines are considered as possible mediators for initiating inflammatory events in the complex coordination network of pulmonary cells [3, 7, 37].

The present study has investigated the potential mutagenic effects of asbestos-activated alveolar macrophages in Cocultured fibroblasts. With the aim of proposing an alternative in vitro mutagenesis test, commercially available rat alveolar NR8383 cells were selected as macrophages in our model. The Big Blue rat fibroblast cell line was used as a target cell to assess mutagenic effects. 

The asbestos fibre tested in the coculture model was a crocidolite sample, which has been shown previously to be mutagenic in transgenic mice [9]. This sample was a mixture of short and long (>5 *μ*m) fibres. As observed using optical and scanning electron microscopy, 24-hour contact of crocidolite with NR8383 macrophages produced both efficient and unsuccessful phagocytoses, depending on fibre-size (data not shown). 

The capacity of this crocidolite sample to stimulate ROS generation in NR8383 was investigated by chemiluminescence and fluorescence methods. No oxidative activity in NR8383 exposed to crocidolite was detected, but the two methods provided evidence of high production of oxidative species following exposure to zymosan. Zymosan has been used previously to demonstrate the capacity of NR8383 cells to produce an oxidative burst, and our results are similar to those reported [21, 38]. There are no available data concerning the effect of crocidolite treatment on NR8383 cells. Similar to our observations, no increase in ROS production was observed in primary cultures of rat alveolar macrophages exposed to crocidolite (tested at 15 *μ*g/cm^2^), as detected by cytochrome *c* reduction [39]. In a previous study, using a chemiluminescence method, two asbestos fibres, erionite and mordenite, were demonstrated to be capable of inducing a brief increase of oxidative species in NR8383 cultures, but at relatively high particle concentrations (about 300 particles/cell) [40]. When tested with another phagocyte type, the human primary blood polymorphonuclears, crocidolite was shown to produce a brief generation of free radicals at a concentration corresponding to 250 *μ*g/10^6^ cells [41, 42]. Human monocytes can similarly generate ROS after contact with amosite, chrysotile, ceramic, wool, or glass fibres [17, 18, 43]. In their coculture model, Kienast et al. used primary human blood monocytes exposed to chrysotile (50 and 100 *μ*g/10^6^ cells) as a “generator” system of reactive oxidative intermediates [17]. Consequently, both published data and our results indicate a poor capacity of rat alveolar macrophages to generate oxidative species when exposed in vitro to crocidolite. In contrast, primary human blood monocytes have been shown to be reactive with a wide variety of mineral fibres, including crocidolite. When detected, the release of ROS is typical of an oxidative burst (lasting no more than 30 minutes) and is obtained with high concentrations of fibres. In our chemiluminescent assay, the highest crocidolite concentration inducing less than 20% mortality in NR8383 after 3 hours was 60 *μ*g/cm^2^, which was equivalent to 75 *μ*g/10^6^ cells or about 50 fibres/cells. At this level, the amount of fibres can be considered as a large excess in our assays. Crocidolite concentrations up to 200 *μ*g/cm^2^ were tested but no oxidative activity was detected (data not shown).

The study of the gene expression of various inflammatory factors in NR8383 and Big Blue was a different approach used to assess the capacity of crocidolite to activate NR8383 and its effect on the cocultivated fibroblasts. A system of separated coculture of NR8383 and Big Blue cells was used, similar to the system described by Kienast et al., which allows selective exposure of phagocytes to fibres [17]. NR8383 were exposed first to high concentrations of crocidolite or zymosan (up to 60 *μ*g/cm^2^) for a short time (3 hours), conditions that normally favour the release of ROS from phagocytes. Considering the possibility that NR8383 activation by crocidolite is triggered less quickly than an oxidative burst, the duration of treatment with crocidolite was extended to 24 hours but at lower concentrations (3 and 15 *μ*g/cm^2^) to avoid cytotoxic effects. Both crocidolite and zymosan induced a significant elevation of iNOS, IL 1*β*, and TNF*α* expression in NR8383, indicating their capacity of activation. However, 24-hour treatment with crocidolite was necessary to reach levels of iNOs and IL1-*β* expression equivalent to those obtained after only 3 hours of zymosan exposure. The level of TNF*α* expression induced by crocidolite remained low, whatever the duration of exposure was. These results indicate that NR8383 activation by crocidolite is more of a progressive process than a “burst effect” as induced by zymosan.

The capacity of crocidolite to stimulate the expression of TNF*α*, IL 1*β*, and iNOS in alveolar macrophages has been demonstrated in vivo and in vitro previously in [13, 17, 36, 39, 44]. In particular, primary rat alveolar macrophages exposed in vitro to the same dose range used in our study (15 *μ*g/cm^2^) produced a progressive and persistent production of IL1 and TNF*α* (in up to 14 days of exposure). So, it was not surprising to obtain a higher level of IL1-*β* gene expression after 24 hours than after 3 hours of crocidolite exposure. The low levels of TNF*α* expression in NR8383 after crocidolite exposure could be explained by the inability of NR8383 cells to generate ROS, as it has previously been shown that the presence of free radicals are important in stimulating TNF*α* release from rat alveolar macrophage exposed to asbestos fibres [45].

As a secondary effect, crocidolite-exposed NR8383 increased the gene expression of iNOS and SOD2 in the cocultivated Big Blue fibroblasts. The significant up-regulation of iNOS detected in Big Blue is important to note, because it suggests the formation of potentially genotoxic nitrogen species (NO and peroxynitrite) during the treatment. However, expression levels of iNOS remained quite low compared to those obtained after zymosan exposure, even when the treatment time was extended to 24 hours. Interestingly, the increase in SOD2 expression in Big Blue after 24-hour exposure to crocidolite could reflect ROS formation in fibroblasts. In contrast to the effect of zymosan, no stimulation of the Il 6 gene was observed from crocidolite exposure in Big Blue. The capacity of monocytes exposed to chrysotile for inducing Il 6 cytokine expression in cocultivated bronchial epithelial cells has been reported [20]. Crocidolite probably induced different secondary effects in our coculture model due to a lack of TNF*α* production by NR8383. 

Mutagenesis assays were first conducted under separated coculture conditions. In their coculture model, Kienast et al. exposed human blood monocytes to chrysotile for 1, 3, 24, and 48 hours [17]. According to the authors, the main DNA strand lesion in cocultivated bronchial epithelial cells occurred within 1 hour of exposure. In our study, based on the assumption that NR8383 activation by crocidolite is progressive, a long-term treatment with crocidolite was preferred (72 hours). In addition, genomic mutations are normally more stable over time than DNA strand lesions. In parallel, we also tested crocidolite in a mixed coculture system to increase the chance of oxidative species with a very short life to react with Big Blue cells. For these tests, treatment time was reduced to 24 hours to limit cytotoxic side effects. Zymosan was also tested in the mixed coculture system, under the short treatment conditions (3 hours) that induced the oxidative burst in NR8383. Spontaneous and ENU-induced mutation frequencies obtained in Big Blue monocultures were within the same range as those reported previously in [29]. In contrast, no induction of mutagenesis was observed in Big Blue cells after exposure with crocidolite in mixed or separated NR8383 cocultures. This result is probably explained by the low potential of crocidolite for inducing ROS production from NR8383. Up-regulation of iNOS, an SOD2 expression in Big Blue induced by crocidolite that could suggest some free radical generation, was not associated with mutagenic effects in our coculture system. However, zymosan was shown to increase Big Blue mutant frequency in mixed cocultures after 3-hour exposure (up to a 2.4-fold increase relative to the control cocultures). This result was consistent with the efficiency of zymosan in inducing significant release of ROS from NR8383. Other studies have already demonstrated that ROS and/or RNS production from macrophages activated with lipopolysaccharide and interferon induces mutations in cocultivated cells [46, 47]. The possibility that macrophage activation by zymosan particles can produce mutagenic effects in neighbouring cells has not been reported previously. 

In conclusion, the coculture system tested with zymosan provided additional evidence that free radical production by activated macrophages can be responsible for genotoxic effects. However, no such effect was obtained with a crocidolite sample, which had been found to induce genomic point mutation in vivo [9]. Consequently, the mutagenesis assay based on the coculture of NR8383 and Big Blue cannot be used as an alternative in vitro method to assess the mutagenic properties of asbestos fibres. NR8383 activation by crocidolite is probably insufficient to induce in vitro mutagenic events in neighbouring cells. Human blood monocytes would have been phagocytes of interest in the purpose of this study, as data from literature indicate that they generate ROS in contact with a variety of mineral fibres. However, this primary cell type is difficult to obtain routinely and may not be suitable for an alternative in vitro mutagenesis assay. 

## Figures and Tables

**Table 1:** Details of primer sets used.

Primers	GenBank #		Sequences (5′–3′)
TNF-*α*	X66539	Sense	AAA TGG GCT CCC TCT CAT CAG TTC
		Antisense	TCT GCT TGG TGG TTT GCT ACG AC
IL1-*β*	M98820	Sense	CAC CTC TCA AGC AGA GCA CAG
		Antisense	GGG TTC CAT GGT GAA GTC AAC
iNOS	L12562	Sense	CAT TGG AAG TGA AGC GTT TCG
		Antisense	CAG CTG GGC TGT ACA AAC CTT
IL6	E02522	Sense	TCC TAC CCC AAC TTC CAA TGC TC
		Antisense	TTG GAT GGT CTT GGT CCT TAG CC
SOD2	NM_017051	Sense	TAAGGAGCAAGGTCGCTTACA
		Antisense	TAAGGAGCAAGGTCGCTTACA
*β*-actin	V01217	Sense	AAG TCC CTC ACC CTC CCA AAA G
		Antisense	AAG CAA TGC TGT CAC CTT CCC

**Table 2:** DCF fluorescence in particle-exposed NR8383 analysed by flow cytometry.

Treatment	Mean fluorescence ± SD^(1)^
Control	77.1 ± 5,9
Crocidolite 1 *μ*g/cm^2^	71.5 ± 2,9
Crocidolite 5 *μ*g/cm^2^	65.9 ± 13,5
Crocidolite 10 *μ*g/cm^2^	65.7 ± 15,2
Zymosan 10 *μ*g/cm^2^	236.5 ± 19,1*

^(1)^Result obtained from three experiments.

*Statistically different from control values (*P* < .05).

**Table 3:** Cytotoxicity and mutation frequencies in Big Blue cells following various treatments in mono- or coculture with NR8383 cells.

Treatment and culture condition		% cell mortality (Mean ± SD)	PFU per assay	Mutant plaques	MF (×10^−5^)	Group average MF (×10^−5^± SD)
ENU, monoculture, 30 min	Control	1.2 ± 0.5	375000	32	8.5	9.6 ± 1.0
			350000	36	10.3	
			455000	46	10.1	

	100 *μ*g/mL	2.3 ± 1.4	285000	133	46.7	41.8 ± 8.7*
			553333	176	31.8	
			338330	159	47.0	

	500 *μ*g/mL	2.2 ± 0.5	223000	208	93.3	83.3 ± 9.8*
			368330	306	83.1	
			203666	150	73.6	
						

Crocidolite separated coculture, 72 h	Control	4.1 ± 1.9 (BB); 5.4 ±2.3 (NR)	423334	47	11.1	12.9 ± 1.6
			860333	102	11.9	
			868333	122	14.0	
			566667	79	13.9	
			675000	79	11.7	
			573333	86	15.0	

	3 *μ*g/cm^2^	5.8 ± 2.0 (BB); 7.9 ±2.0 (NR)	349000	25	7.2	8.7 ± 1.7
			477333	41	8.6	
			506166	53	10.5	

	15 *μ*g/cm^2^	3.6 ± 1.8 (BB); 19.6 ±1.3* (NR)	1090000	127	11.7	13.2 ± 2.2
			786667	77	9.8	
			445000	70	15.7	
			723333	104	14.4	
			567667	82	14.4	
			616667	81	13.1	

Crocidolite, mixed coculture, 24 h	Control	8.2 ± 0.9	303350	63	20.8	19.0 ± 2.3
			303350	57	18.8	
			301650	68	22.5	
			460000	79	17.2	
			501650	83	16.5	
			388350	71	18.3	

	10 *μ*g/cm^2^	15.0 ± 1.4*	246650	43	17.4	18.5 ± 4.4
			246650	63	25.5	
			331650	71	21.4	
			475333	61	12.8	
			692333	122	17.6	
			323350	52	16.1	

Zymosan, mixed coculture, 3 h	Control	7.1 ± 2.0	468333	69	14.7	13.0 ± 1.5
			248000	30	12.1	
			593333	72	12.1	

	15 *μ*g/cm^2^	6.4 ± 0.8	525000	82	15.6	18.3 ± 2.9*
			656667	118	18.0	
			393334	84	21.4	

	30 *μ*g/cm^2^	7.0 ± 0.9	471667	83	17.6	17.4 ± 1.4*
			833334	156	18.7	
			600000	96	16.0	

	60 *μ*g/cm^2^	7.7 ± 1.8	465000	134	28.8	30.8 ± 7.0*
			574167	144	25.1	
			513333	198	38.6	

PFU: plaque forming unit; MF: mutant frequency; BB: Big Blue; NN: NR8383; *Statistically different from control values (*P* < .05).
